# The Ophthalmology of the Poets

**Published:** 1896-02-29

**Authors:** 


### The Ophthalmology of the Poets.

### II.—SHAKESPEARE, TENNYSON, AND SCOTT.

The poets do not often notice hazel eyes, but we recall at least two occasions on which mention of them is made. Shakespeare apparently connects them with a quarrelsome nature, for in "Romeo and Juliet" Mercutio says to Benvolio, "Thou, why thou wilt quarrel with a man that hath a hair more, or a hair less, in his beard, than thou hast? Thou wilt quarrel with a man for cracking nuts, having no other reason but because thou hast hazel eyes. What eye, but such an eye, would spy out such a quarrel? Thy head is as full of quarrels as an egg is full of meat."

Tennyson, in "Locksley Hall," describes Amy as having hazel eyes—though he does not seem to have thought less of her on that account.

A point about eyes on which poets constantly dwell is their brightness, or otherwise. The eye can only be bright while the circulation within it is active; in low conditions of vitality there is always a diminution of lustre, while it is completely lost in death.

Scott's minstrel, an old and feeble man, is described as having "a faded eye," and the effect of a temporary stimulus on the eye of a dying man is seen in "Marmion":—

> The war, that for a space did fail,
> New trebly thundering swelled the gale,
> And—Stanley! was the cry:—
> A light on Marmion's visage spread,
> And fired his glazing eye;
> With dying hand, above his head,
> He shook the fragment of a blade,
> And shouted "Victory!"
> "Charge, Chester, charge! On, Stanley, on!"
> Were the last words of Marmion.

In "As You Like It" Shakespeare describes Touchstone, the clown, as having a "lack-lustre eye," and the same phrase is used by Tennyson regarding an old man; and Browning speaks of "the lion, when age dims his eyeball."

On the other hand, sparkling or glancing eyes have been praised by nearly every poet since poets first existed. Longfellow, it is true, is an exception. He disliked sparkling eyes, saying that those only are beautiful which, like the planets, have a steady, lambent light—are luminous, not sparkling.

Shakspeare speaks of women's eyes "that sparkle still the right Promethean fire." Moore sings often of such: "Lesbia hath a beaming eye," "Fatal's the glance of Kate Kearney," and so on; while no simile is more often used than that comparing eyes to stars, as in Shakespeare:—

> What stars do spangle heaven with such beauty
> As those two eyes become that heavenly face?

And again:—

> Stars, stars, and all eyes else dead coals.

Next in favour to the stars come the sun and diamonds, as in Henry VI.:—

> Those eyes that now are dimmed with death's black veil
> Have been as piercing as the midday sun.

And, again, "her sun-bright eye" and "thine eyes would emulate the diamond."

Comparisons with the eyes of animals have been common in all ages, since Homer described Juno as "ox-eyed," some such comparisons being complimentary, and some much the reverse. In the East especially these similes are used. In a Turkish love song which has been translated, the lover addresses his mistress as "stag-eyed," and "eyes like a gazelle" is a favourite expression. These various terms no doubt aim at describing the large clear eye, moving slowly in its orbit, and having a mild or gentle expression. The modern or Western description of the same characteristics is seen in Tennyson's "Œnone," where he speaks of the goddess Pallas Athene as having "full and earnest eyes," and in Lewis Morris's description of the same divinity:—

> A fair pale form drew near
> With front severe, and wide blue eyes which bore
> Mild wisdom in their gaze.

Shakespeare often compares human eyes to those of the lower animals, but generally to indicate their keen, quick glance, for the animals named are birds or beasts of prey, whose small, quickly-moving eyes are very different from the large, gentle eyes of the ruminant animals mentioned above. Such expressions as "an eagle-sighted eye," "a hawking eye," a "ferret and fiery eye" are found in Shakespeare, and "falcon-eyed" in Tennyson.

So far I have spoken of the eye in its normal anatomical aspect. Much might be said of the poets' references to eyes from a pathological standpoint—from Shakespeare's mention of a "green sarcenet flap for a sore eye" down to the modern bard's lyric, lately so popular, celebrating the occurrence of a case of bilateral palpebral and subconjunctival ecchymosis in a gentleman who was simply "walking down Bethnal Green"—but this paper has reached its limits. It cannot be closed better than with the words of the witty Phœbe in "As You Like It," which are, to my thinking, the most beautiful reference to the eye in English poetry:—

> Thou tell'st me there is murther in mine eye;
> 'Tis pretty, sure, and very probable,
> That eyes, that are the frail'st and softest things,
> Who shut their coward gates on atomies,
> Should be called tyrants, butchers, murtherers!
> Now I do frown on thee with all my heart:
> And if mine eyes can wound, now let them kill thee;
> Now counterfeit to swoon; why, now fall down;
> Or, if thou canst not, O, for shame, for shame,
> Lie not, to say mine eyes are murtherers.
> Now show the wound mine eye hath made in thee;
> Scratch thee but with a pin, and there remains
> Some scar of it; lean upon a rush
> The cicatrice and palpable impressure
> Thy palm some moment keeps; but now mine eyes,
> Which I have darted at thee, hurt thee not;
> Nor, I am sure, there is no force in eyes
> That can do hurt.

